# Rapid detection of *Mycobacterium tuberculosis* in children using blood and urine specimens

**DOI:** 10.1590/0037-8682-0051-2020

**Published:** 2020-09-25

**Authors:** Juliana Figueirêdo da Costa-Lima, Lílian Maria Lapa Montenegro Pimentel, Fabiana Cristina Fulco Santos, Marcela Pereira Salazar, Rafael Silva Duarte, Fernanda Carvalho de Queiroz Mello, Haiana Charifker Schindler

**Affiliations:** 1 Instituto Aggeu Magalhães/Fundação Oswaldo Cruz, Laboratório de Imunoepidemiologia, Departamento de Imunologia, Recife, PE, Brasil.; 2 Universidade Federal do Rio de Janeiro, Instituto de Microbiologia Paulo Góes, Laboratório de Micobactérias, Rio de Janeiro, RJ, Brasil.; 3 Universidade Federal do Rio de Janeiro, Instituto de Doenças do Tórax, Faculdade de Medicina, Rio de Janeiro, RJ, Brasil.; 4 Universidade Federal de Pernambuco, Hospital das Clínicas, Departamento Materno Infantil, Recife, PE, Brasil.; 5 Universidade Federal do Rio de Janeiro, Programa de Pós-Graduação Stricto Sensu em Clínica Médica, Rio de Janeiro, RJ, Brasil.

**Keywords:** Childhood tuberculosis, Diagnosis, Blood, Urine, PCR, STNPCR

## Abstract

**INTRODUCTION:**

Laboratory and clinical features of childhood tuberculosis (TB) are non-specific and establishing an accurate diagnosis remains a challenge. This study evaluated a Single tube nested-PCR (STNPCR) to detect genomic DNA of *Mycobacterium tuberculosis* complex in blood and urine.

**METHODS:**

Biological samples were obtained from children (<15 years old) with clinical suspicion of pulmonary and extrapulmonary TB at public hospitals in Recife-Pernambuco, Brazil. Cultures yielded negative results in a majority of childhood TB cases, which are generally paucibacillary. A set of clinical, epidemiological, radiological, and laboratory criteria with evident clinical improvement after anti-TB treatment were frequently used to define childhood TB cases.

**RESULTS:**

Ninety children with clinical suspicion were enrolled in this study (44 with TB and 46 without TB). The pulmonary TB group had 20 confirmed cases and 46 negative controls, while the extrapulmonary TB group had 24 confirmed cases. The STNPCR showed sensitivities to pulmonary and extrapulmonary TB of 47.4% and 52.2% (blood) and 38.8% and 20% (urine), respectively. Considering the low performance of STNPCR on separate samples, we decided to perform a combined analysis (parallel sensitivity analysis) of the results from blood and urine samples. The parallel sensitivity increased to 65% in blood and 62.5% in urine. The specificity in both samples ranged from 93.5-97.8%.

**CONCLUSIONS:**

Although STNPCR showed moderate sensitivity, the specificity is high; therefore, the test can be used as an auxiliary tool to diagnose TB in children. It is a rapid test that demonstrated better performance than other diagnostic tests in paucibacillary samples as it does in childhood tuberculosis.

## INTRODUCTION

According to the World Health Organization (WHO), 6.9% of all tuberculosis (TB) cases were notified among children (2016). The WHO estimated that more than 200,000 (16%) deaths from TB occurred among HIV-negative^1^ children, which makes TB one of the top 10 causes of death among children^2^. Brazil is ranked as 25^th^ among 30 countries with the highest prevalences of TB, which are responsible for 82% of TB cases worldwide and 75% of childhood TB cases^1^ ^,^ ^3^. According to the Brazilian Notification of Injury Information System (*Sistema de Informação de Agravos de Notificação - SINAN*), in 2018, more than 94,000 cases of TB were registered in Brazil, where almost 7,900 confirmed cases occurred among patients younger than 19 years^4^.

Childhood TB does not have accurate epidemiological data, because of difficulty in establishing a diagnosis^5^. Most cases of TB in this age group are pulmonary and only 20% of total cases are extrapulmonary. However, the more severe forms are extrapulmonary and occur frequently among children as miliary TB and TB meningitis (mainly among children younger than 6 years). Peripheral lymph nodes and TB meningitis are the most frequent manifestations among children^6^ ^-^ ^8^.

The diagnosis of TB is based on the identification of *Mycobacterium tuberculosis* bacilli through smear microscopy, culture, or Xpert^®^ MTB/RIF assay^1^ ^,^ ^6^. However, the diagnosis of TB in childhood is a big challenge, as it has no accurate gold standard diagnostic method^9^ ^-^ ^12^. Conventional techniques used in the diagnosis of adult patients present low sensitivity and specificity when applied to children. Bacteriological confirmation is not possible, because childhood TB is paucibacillary. Thus, very frequently, anti-TB treatment is initiated with no bacteriological confirmation^13^ ^-^ ^15^.

Children younger than 10 years normally have no expectoration and have clinical signs and radiological examination findings that are complex to interpret. In this paucibacillary group, conventional diagnostic methods have poor sensitivity and specificity. The younger the child, the more unspecific the clinical symptoms are and the higher the risk of gravity of TB and death. The opposite also occurs: the older the child is, the more similar is the disease to the adult form^16^ ^,^ ^17^. 

There is almost no validated score for the diagnosis of childhood TB. In 2002, the Brazilian Ministry of Health (MH) recommended the use of a scoring system, based on clinical, radiological, epidemiological criteria and tuberculin skin test results to diagnose pulmonary TB in children and adolescents^6^ ^,^ ^18^. The score proposed by the MH of Brazil, which is known to confirm the diagnosis of TB in children and teenagers, is one of the most widely validated worldwide and which has consistent sensitivity and specificity^6^.

Since 2011, the WHO has recommended the use of Xpert^®^ MTB/RIF (Cepheid, CA, USA) as a rapid test for the diagnosis of pulmonary TB and resistance to rifampicin^19^ ^,^ ^20^. More recently, the Xpert MTB/RIF assay was recommended for the diagnosis of pulmonary TB in children. The Xpert^®^ MTB/RIF assay has been evaluated in 13 studies that included a total of 3,347 specimens for the diagnosis of pediatric pulmonary TB, whose sensitivity varied from 55% to 90% for expectorated sputum, from 40% to 100% for induced sputum, and from 40% to 100% for gastric lavage or aspirate^21^. The MH of Brazil also recommends the use of molecular rapid tests for suspected cases of pulmonary TB when scores do not confirm the diagnosis^6^.

Molecular methods have been developed for use in the detection of bacilli and confirmation of disease. The use of polymerase chain reaction (PCR) assays has been proposed in a few studies to increase the sensitivity and specificity of the diagnosis of childhood tuberculosis^6^ ^,^ ^22^. Generally, they have high sensitivities and specificities using clinical samples and produce rapid results^9^ ^,^ ^10^ ^,^ ^23^ ^-^ ^25^. These techniques detect a specific genome DNA region in the microorganism. 

Thus, it is necessary to evaluate a new system for the detection of *M. tuberculosis* among children, particularly because of the limitations of conventional diagnostic methods. Based on the difficulty of diagnosis of childhood TB, among patients who do not have sputum expectoration, this study proposed the use of non-invasive samples, blood and urine, to detect the DNA of *M. tuberculosis* by single tube PCR (STNPCR)^23^ and evaluated it in comparison with other conventional tests in clinically suspected pulmonary and extrapulmonary TB. Blood and urine can be collected from outpatients regardless of the site of infection^26^.

The aim of the present study was to evaluate the new system of STNPCR for the detection of *Mycobacterium tuberculosis* DNA in alternative clinical specimens (blood and urine) via minimally invasive methods, other than sputum collection from children and adolescents with clinically suspected pulmonary or extrapulmonary TB.

## METHODS

### Study design, population, and setting

The present prospective study was conducted among 90 children with an initial clinical suspicion of pulmonary or extrapulmonary TB, up to 15 years of age, both sexes, and who spontaneously sought out hospitals or primary health care centers in Recife-Pernambuco, Brazil, considered a high-risk area for TB. It was developed at the laboratory of Immunoepidemiology of the Aggeu Magalhães Institute/Oswaldo Cruz Foundation (IAM/FIOCRUZ), between September 2009 and April 2014. 

### Classification of groups

All patients were prospectively followed and classified into two groups (active TB and not TB) using clinical, laboratorial, and therapeutic response criteria^27^ according to the American Thoracic Society guidelines^28^, WHO guidelines^1^, and the Brazilian MH guidelines^6^. The attending physicians at hospitals made the final double-blind diagnoses. 

### Eligibility criteria

**Active TB (pulmonary and extrapulmonary):** Patients with clinical symptoms or radiological images compatible with TB, epidemiological history of contact with a person with bacilipherous TB, with or without isolation of *M. tuberculosis* on acid-fast bacilli (AFB) or on culture, or with clinical improvement consequent to specific anti-TB treatment (reference standard used).

**Not TB:** Patients with a diagnosis of another disease other than TB and without isolation of *M. tuberculosis*.

### Biological samples

All children had one sample of total blood (3-5 mL collected in a vacuum tube with anti-coagulant EDTA) and/or three samples of urine (10 mL/day, on three consecutive days), which were merged at the laboratory to be analyzed as an unique sample on STNPCR. All samples were collected before the initiation of specific treatment.

### Blood - Isolation of peripheral blood mononuclear cells and plasma

Peripheral blood mononuclear cells (PBMCs) and plasma were separated from whole blood at room temperature by the density gradient method using Ficoll-Paque^TM^ Plus (GE Healthcare, Sweden). The erythrocytes were discarded and the PBMCs and plasma layers were separated to be used in DNA extraction and afterwards in STNPCR. 

### Urine decontamination

Urine samples were decontaminated using the Petroff’s method with 4% NaOH^29^ ^,^ ^30^.

### DNA extraction

DNA extraction was performed using the commercial QIAmp DNA mini kit (QIAGENGmbH, Hilden, Germany), as per the manufacturer’s recommendation. The DNA was extracted from PBMC, plasma, and urine samples. For all DNA extractions, a negative control tube (with TE buffer and no DNA template) were used to evaluate possible cross-contamination. 

### Molecular STNPCR system

The STNPCR system was based on the principles put forth by Abath et al.^31^ and Costa-Lima et al.^23^ using an IS6110 insertion sequence (GenBank accession no. X52471) as a target to detect DNA from *M. tuberculosis* complex. The set of outer primers used were TJ5 (5′-CCGCAAAGTGTGGCTAAC-3′) and TJ3 (5′-ATCCCCTATCCGTATGGTG-3′) with an amplified fragment of 409 bp. The inner primers were OLI5 (5′-AACGGCTGATGACCAAAC-3′) and STAN3 (5′-GTCGAGTACGCCTTCTTGTT-3′), amplifying a 316 bp fragment^32^. These sets of primers were previews used on a conventional nested PCR system and on STNPCR of blood and urine^23^ ^,^ ^33^.

### Ethical considerations

The study protocol was approved by the ethics committee (CAAE 08381812.4.00005190) of IAM/FIOCRUZ. Written informed consent was obtained from all guardians or legal representatives of each child who agreed to participate in the research and authorized the collection of clinical samples.

### Statistical analysis

The database was prepared using SPSS Statistics version 20.0.0 (IBM Corp., Armonk, NY, USA), in which all crosstabs and frequencies and other analyses, excluding the sensitivities, specificities, and predictive values (screening tests) were assessed. For screening tests, the free software OpenEpi version 2.3.1 was used.

For statistical analyses, PBMC and plasma samples were considered as unique samples named “blood sample” and their sensitivity, specificity, and predictive values were analyzed in parallel^34^. In the same way, the “blood samples” plus urine samples were evaluated in parallel.

## RESULTS

### Clinical, epidemiological, and laboratorial results

Forty-four (47.3%) children were diagnosed with active TB from a total of 90. The mean age was 7.5 ± 4.9 years (range, 0-15). Only one child was known to have a coinfection of HIV and TB; for the remaining children, HIV tests were not performed. The inpatients (51.6%) were the majority in relation to outpatients. AFB tests were performed among 11 (12.8%) children, with only one positive result (a 14-year-old girl). Regarding culture, 86% of children underwent culture for at least one clinical sample (urine, sputum, pleural fluid, and/or other fluids). Culture test results were positive in only 10 (11%) of them. A majority of cultures were performed on urine (66.7%), with positive results in 6.9% (4/58). Other epidemiological and demographic data are detailed in Table 1. The chi-squared analysis of type of hospital admission, result of skin test, and treatment response, yielded *p* values of 0.005, 0.02 and <0.001, respectively. Each child provided a mean of 2.8 clinical samples for analysis. Most cases were of pulmonary TB, followed by peripheral lymph node TB. All clinical manifestations of TB are detailed in Table 2.

**TABLE 1:** Frequency of clinical and epidemiological characteristics of children (*n* = 90) and their respective prevalence ratio and *p-value* (95% CI) on a Poisson binary regression related to confirmation of TB

Characteristics	TB	Not TB	Prevalence ratio -	*p-value*
			Poisson binary	
			regression (CI)	
**Hospital admission**				
Outpatients	26 (59.1%)	16 (34.8%)		-
Inpatients	18 (40.9%)	30 (65.2%)	1.29 (1.06-1.57)	**0.01**
**Total**	**44**	**46**		-
Sex				
Male	18 (40.9%)	25 (54.3%)		-
Female	26 (59.1%)	21 (45.7%)	0.77 (0.52-1.17)	0.23
**Total**	**44**	**46**		-
**Age (by years)**	**44**	**46**	1.02 (1-1.05)	0.053
**Age (by age groups)**				
From 0 to 5 years	10 (22.7%)	22 (47.8%)	-	
From 6 to 10 years	13 (29.5%)	8 (17.4%)	-	
From 11 to 15 years	19 (43.3%)	14 (30.4%)	1.15 (1.02-1.31)	**0.029**
No age (by years) information	2 (4.5%)	2 (4.4%)	-	-
**Scar of BCG vaccine**				
Yes	31 (70.5%)	26 (56.5%)	-	-
No	1 (2.3%)	3 (6.5%)	0.73 (0.47-1.14)	0.171
Not verified	12 (27.2%)	17 (37%)	-	-
**Total**	**44**	**46**	**90**	-
**Type of contact with bacillipherous TB**				
Contact with bacillipherous	16 (55.2%)	19 (61.3%)	-	-
No contact with bacillipherous	13 (44.8%)	10 (32.3%)	0.37 (0-0)	**<0.001**
Unable to assess	0 (-)	2 (5.4%)	-	-
**Total**	**29**	**31**	**60**	-
**AFB results (all samples)**				
Positive†	1 (1.1%)	0	-	-
Negative	10 (11.1%)	0	0.61 (0.45-0.83)	**0.002**
Not realized	33 (87.8%)	46 (100%)	-	-
**Total**	**44**	**46**	**90**	-
**Culture (all samples)**				
Positive	2 (4.5%)	1 (2.2%)	-	-
Negative	15 (34.1%)	18 (39.1%)	0.81 (0.46-1.42)	0.46
Not realized	27 (61.4%)	27 (58.7%)	-	-
**Total**	**44**	**46**	**90**	-
**Result of skin test**				
< 10 mm	13 (29.5%)	15 (32.6%)	-	-
> 10 mm	18 (41%)	5 (10.9%)	0.73 (0.57-0.93)	**0.013**
Not realized	13 (29.5%)	26 (56.5%)	-	-
**Total**	**44**	**46**	**90**	-
**Clinical form of TB**				
Pulmonary TB	20 (42.6%)	0	-	-
Extrapulmonary TB	24 (51.1%)	0	-	-
Not TB	0	46 (100%)	-	-
**Total**	**44**	**46**	**90**	-

*AFB and culture performed at IAM/FIOCRUZ, independent of results from the reference laboratory; **†** Patient had result for AFB of one cross; **AFB:** acid fast bacilli; **BCG:** Bacillus Calmette-Guérin; **TB:** tuberculosis

**TABLE 2:** Clinical forms of tuberculosis among children.

Clinical form	Frequency	Percentage
Pulmonar	20	45.5%
Pleural	3	6.8%
Peripheral lymph node	7	15.9%
Meningoencephalitis	2	4.5%
Bone	2	4.5%
Articular	1	2.3%
Cutaneous	1	2.3%
Abdominal	2	4.5%
Pericardial	1	2.3%
Intestinal	1	2.3%
Miliary	1	2.3%
Other	3	6.8%
**Total**	**44**	**100%**

### Single tube nested PCR

This study evaluated the performance of STNPCR in each clinical sample collected: PBMC, plasma, and urine. Regarding loss criteria (insufficient material, hemolyzed blood, or other), four patients had no blood sample, in two other patients it was not possible to separate PBMCs, and eight children did not have urine samples. The sensitivities of tests varied from 26% to 50% and specificities from 94% to 100% (Table 3). Calculating in parallel, the sensitivity of “blood sample” plus urine was above 60%. The results for each sample analyzed alone and in parallel are detailed in Table 3. According to the clinical form of TB, accuracy was evaluated via STNPCR on “blood samples” alone and with urine (20 with pulmonary TB, 24 with extrapulmonary TB, and 46 with no TB), calculated in parallel (Table 3). Four patients had no blood (neither PBMC nor plasma) collected, two others had no PBMCs isolated from plasma, and eight children had no urine sample, all of whom were included in the study with only one sample (blood or urine). The positive and negative predictive values for this population (*n=*90) were 90.3% (28/31) and 72.9% (43/59), for all biological samples analyzed in parallel.

**TABLE 3:** Performance of STNPCR by type of biological sample and among patients with and without TB (pulmonary and extrapulmonary).

Clinical samples	Nº of samples	Patients with and without TB (all clinical forms)	
		Sensitivity	Specificity
PBMC	84	39%	95.4%
		25.7, 54.3	84.5, 98.7
		(16/41)	(41/43)
Plasma	86	26.2%	100%
		15.3, 41.1	92, 100
		(11/42)	(44/44)
Blood sample (PBMC plasma)	86	50%	95.5%
		35.5, 64.5	84.9, 98.7
		(21/42)	(42/44)
Urine	82	28.2%	97.7%
		16.5, 43.8	87.9, 99.6
		(11/39)	(42/43)
Blood sample + urine	90	63.6%	93.5%
		48.9, 76.2	82.5, 97.8
		(28/44)	(43/46)
Clinical samples	**Patients (n)**	**Pulmonary TB (95% CI)**	
		**Se**	**Sp**
Blood samples*	63	47.4%	95.5%
		(27.3, 68.3)	(84.9, 98.7)
		9/19	42/44
Urine samples	62	38.8%	97.7%
		(19.2, 59)	(87.9, 99.6)
		7/19	42/43
Blood and urine* *per* patient	66	65%	93.5%
		(43.3, 81.9)	(82.5, 97.8)
		13/20	43/46
Clinical samples	**Patients (n)**	**Extrapulmonary TB (95% CI)**	
		**Se**	**Sp**
Blood samples*	67	52.2%	95.5%
		(33, 70.8)	(84.9, 98.7)
		12/23	42/44
Urine samples	65	20%	97.8%
		(8.1, 41.6)	(88.4, 99.6)
		4/20	44/45
Blood and urine* *per* patient	70	62.5%	93.5%
		(42.7, 78.8)	(82.5, 97.8)
		15/24	43/46

*Patient was considered positive when at least one sample yielded positive results on STNPCR. Se: Sensitivity; Sp: Specificity; CI: confidence interval; STNPCR: single tube nested PCR; PBMC: peripheral blood mononuclear cell.

Fifty-six patients had their diagnoses confirmed via cultures performed at the Central Laboratory of Pernambuco, which is a reference center for the diagnosis of TB. The other 34 patients (with no microbiological test confirmation), had TB diagnoses confirmed by therapeutic empirical tests. From this group (therapeutic empirical test) of 34 patients, thirty responded positively and 4 had no response. The four children who had no responses to specific treatment also presented negative PCR results. In addition, all 30 who had responded to specific treatment had positive PCR results (Table 4). 

**TABLE 4:** Accuracy of treatment response *versus* STNPCR result among patients (“blood sample” and urine samples).

tSTNPCR	Treatment response		
results			
	Yes	No	Total
Positive	20	0	20
Negative	10	4	14
Total	30	4	34
**Parameter**	**Estimate**	**95% CI**	
Sensitivity	66.7%	(48.8, 80.8)	
Specificity	100%	(51, 100)	
**Treatment response**	**TB**	**Not TB**	
Yes	30 (68.2%)	0 (-)	
No	0 (-)	4 (8.7%)	
Diagnosed by microbiologic tests	14 (31.8%)	42 (91.3%)	
Total	44 (100%)	46 (100%)	

**CI:** confidence interval; **STNPCR:** single tube nested PCR; **TB:** tuberculosis.

## DISCUSSION

In pediatric samples, it is not easy to confirm the presence of bacilli, similar to patients with extrapulmonary TB and TB-HIV coinfection, because they are paucibacillary. These groups, including cases of drug resistant TB, are responsible for the increase in morbidity and mortality due to TB in developing countries^35^.

Regarding samples of reference in adults, sputum is not easy to collect in children because they frequently swallow rather than expectorate it^10^. Some studies advise the use of induced sputum instead for children with pulmonary TB^10^ ^,^ ^20^ and the gastric lavage for extrapulmonary TB^36^. In addition, both of these biological specimens are invasive samples and are not collected at a primary care health center^20^ ^,^ ^36^. On the other hand, one of the best ways to reduce the mortality due to childhood TB is to develop tests that could be run on accessible clinical specimens such as urine and blood^23^ ^,^ ^37^.

A control strategy for TB, particularly in regions with high endemicity, is to develop diagnostic tests which are rapid, sensitive, specific, and inexpensive for use in public health service. It would provide better detection of cases associated with effective treatment, leading to decreased transmission and cases of drug resistant TB. Nucleic acid amplification tests for the diagnosis of pediatric TB could reduce the mortality rate by 6.8%^37^. 

The new technology, Xpert MTB/RIF assay, which should replace smear microscopy at Primary Health Care Centers^20^, is already being used in several countries with a high TB burden. The Xpert MTB/RIF assay yields similar results to the findings of this study on blood and urine collected from children with pulmonary TB^20^. The major disadvantage of Xpert MTB/RIF assay in children is the lowest sensitivity and specificity in respiratory or other samples^38^. In STNPCR, blood and urine are validation samples and can detect both pulmonary and extrapulmonary TB with similar accuracy. Other studies used these samples to detect TB by PCR and showed the importance of using them^23^ ^,^ ^26^ ^,^ ^33^ ^,^ ^39^ ^-^ ^41^ and assisting the diagnosis of TB cases. 

The culture only confirmed TB cases in 11% of pediatric patients. Besides that, culture takes too long to yield results, taking up to eight weeks^8^. Instead, STNPCR can yield test results in up to one day (from sample collection to result) or fewer, demonstrating that it can be used as an auxiliary tool for early diagnosis in children. Generally, for childhood TB, clinical samples are considered as reference standards if the culture yields negative results and the anti-TB therapy can be initiated based on clinical evidence^8^ ^,^ ^21^.

For negative bacteriological cases associated with a strong clinical suspicion of the disease, the empirical treatment is initiated in approximately 30% of suspected TB cases^42^. As demonstrated by this study, most patients with active TB who responded to specific treatment had positive results on STNPCR. Considering empirical therapy as the gold standard, STNPCR presented a sensitivity of approximately 67% and a specificity of 100%. This may be evidence that the use of STNPCR in blood and urine samples may improve laboratory confirmation of cases and avoid the initiation of empirical treatment, which alone has a sensitivity ranging from 20 to 80% in adults^43^. In children, when the risk of death by TB is high, it is highly recommended that empirical treatment be initiated, regardless of confirmation by a diagnostic test^14^ ^,^ ^15^ ^,^ ^44^. It is mainly because there does not exist a single diagnostic test with good accuracy for childhood TB. In the present study, almost 12% of non-TB patients could have avoided unnecessary treatment if negative STNPCR results were considered as diagnostic. It can be concluded that the sensitivity and specificity of STNPCR compared with the clinical, laboratory, and epidemiological criteria or with treatment response were statistically the same, ranging from 63.6 to 66.7% and 93.5 to 100%, respectively.

The STNPCR is a molecular test which detects DNA circulating from *M. tuberculosis* complex in paucibacillary samples. Although, as a molecular test, it does not distinguish viable bacteria cells from non-viable cells, or even from free fragments of nucleic acids in samples. Therefore, STNPCR does not differentiate between active and latent TB. Therefore, the clinical features are paramount to confirm childhood active or latent TB^23^ ^,^ ^45^. 

Based on the difficulty of diagnosis of childhood TB among patients who do not expectorate sputum, we tested non or minimal invasive samples to detect *M. tuberculosis*. Blood and urine can be collected from outpatients regardless of the site of infection.

Some evidence shows the presence of DNA fragments circulating in blood and urine^35^. These fragments are derived from cell-free nucleic acids of bacilli and result from breakdown of these microorganisms or dead human cells, which contained bacilli, and go on to circulate in blood. Some of these fragments of DNA pass through the kidney and are excreted in the urine as transrenal DNA^35^.

According to the results, plasma increases by 21% the sensitivity of PBMC when analyzed in parallel as “blood sample”. When the only clinical sample available for collection would be blood, it must be processed by molecular testing using PBMC and plasma separated and analyzed in parallel, together. Eight children had no urine samples because samples were self-collected at the patients’ homes and some of them did not return to the health care service with the biological sample.

Analyzing the three samples (PBMC, plasma, and urine) isolated yielded no statistical difference between their sensitivities. However, the parallel sensitivity of “blood sample” + urine together was higher than that of plasma or urine alone. Among children with difficult diagnostic interpretation, the collection of more than one clinical sample must be considered to increase the sensitivity of STNPCR. Studies have demonstrated that the combination of two or more different clinical samples from the same patient increases^23^ ^,^ ^26^ the performance of STNPCR. 

Related to the clinical type of TB (pulmonary and extrapulmonary), “blood sample” yielded the most positive results on STNPCR. The urine added around 30% of sensitivity to “blood samples” on parallel sensitivity of STNPCR results. Although this biological sample has a low isolated sensitivity, when associated with “blood sample” it increased the global sensitivity of patients test results^23^.

It was observed in the study that sensitivity of urine in all patients was much lower than that of “blood sample”, but not statistically different. Probably, this difference in results depends on the differences in the physiopathology of clinical disease forms. The sensitivities of “blood sample” added to urine sample, analyzed in parallel, tend to be higher than that of only one isolated sample, both for the pulmonary and extrapulmonary groups. There were no renal TB cases; however, this does not mean that positivity on urine samples indicated false-positive results. The STNPCR does not distinguish between bacilli which are integral than fragments of it (free DNA)^35^. 

In genitourinary TB, PCR is probably the most promising method of detecting *M. tuberculosis* ^23^ ^,^ ^46^. In this study, the sensitivity of urine culture corroborates another study that found that culture usually does not yield a sensitivity of more than 40%^46^. Only one positive urine culture for *M. tuberculosis* also yielded positive results on STNPCR. However, the three other urine cultures positive for nontuberculous mycobacteria strain were all-negative on STNPCR for *M. tuberculosis* complex*.*

For childhood TB, it is difficult to use just one reference test to confirm the disease. A set of criteria is used in almost all cases. Thus, the reference test is subjective and the accuracy of STNPCR could be underestimated. Therefore, to better evaluate the performance of STNPCR instead of the limitations of culture, the authors decided to also consider the treatment response as the gold standard to reflect in fact how childhood TB diagnosis is defined. In these groups of patients, the sensitivity found was similar, but the specificity was 100%. The false-negative samples on molecular test can be associated with the paucibacillary nature of samples or with a possible low efficacy on DNA extraction methods which could not minimize the inhibitory factors^47^.

In this analysis, the results demonstrated that STNPCR is exceptionally reliable in confirming TB because the sensitivity found was 60% and the positive predictive value was 100%. However, not only sensitivity and specificity should be considered to implement a new diagnostic tool; the cost and ease of implementation must be well evaluated^48^ ^,^ ^49^. 

The development of better diagnostic methods is a consensus and remains a significant priority for children^10^ ^,^ ^20^. The proposed method has the great advantage of using clinical samples that are available from most children and obtained via minimally invasive methods. Moreover, this system is fast, sensitive, and specific for use in the diagnosis of TB among children with any clinical form of disease. STNPCR is indicated as an auxiliary tool to help confirm TB in children. Therefore, more studies of the cost-effectiveness of using STNPCR are needed to evaluate the possibility of its implementation in public health services. 
